# Quercetin-3-*O*-glucuronide in the Ethanol Extract of Lotus Leaf (*Nelumbo nucifera*) Enhances Sleep Quantity and Quality in a Rodent Model via a GABAergic Mechanism

**DOI:** 10.3390/molecules26103023

**Published:** 2021-05-19

**Authors:** Singeun Kim, Ki-Bae Hong, Kyungae Jo, Hyung Joo Suh

**Affiliations:** 1 Department of Integrated Biomedical and Life Sciences, Graduate School, Korea University, Seoul 02841, Korea; kimsingun@gmail.com; 2 Department of Food Science and Nutrition, Jeju National University, Jeju 63243, Korea; kibae.hong@gmail.com; 3 Transdisciplinary Major in Learning Health Systems, Department of Healthcare Sciences, Graduate School, Korea University, Seoul 02841, Korea

**Keywords:** *Nelumbo nucifera*, sleep duration, non-rapid eye movement sleep, quercetin-3-*O*-glucuronide

## Abstract

Current pharmacological treatments for insomnia carry several and long-term side effects. Therefore, natural products without side effects are warranted. In this study, the sleep-promoting activity of the lotus leaf (*Nelumbo nucifera*) extract was assessed using ICR mice and Sprague Dawley rats. A pentobarbital-induced sleep test and electroencephalogram analysis were conducted to measure sleep latency time, duration, and sleep architecture. The action mechanism of the extract was evaluated through ligand binding experiments. A high dose (300 mg/kg) of the ethanolic lotus leaf extract significantly increased sleep duration compared to the normal group (*p* < 0.01). Administration of low (150 mg/kg) and high doses (300 mg/kg) of the extract significantly increased sleep quality, especially the relative power of theta waves (*p* < 0.05), compared to the normal group. Furthermore, caffeine and lotus leaf extract administration significantly recovered caffeine-induced sleep disruption (*p* < 0.001), and the sleep quality was similar to that of the normal group. Additionally, ligand binding assay using [^3^H]-flumazenil revealed that quercetin-3*-O-*glucuronide contained in the lotus leaf extract (77.27 μg/mg of extract) enhanced sleep by binding to GABA_A_ receptors. Collectively, these results indicated that the lotus leaf extract, particularly quercetin-3-*O*-glucuronide, exhibits sleep quantity- and quality-enhancing activity via the GABAergic pathway.

## 1. Introduction

Insomnia is caused by a wide variety of psychological factors, such as stress, depression, anxiety, and environmental factors, such as caffeine, alcohol, and cigarettes [1]. Pharmacological treatments, such as benzodiazepines, non-benzodiazepines, and antidepressants, are used to suppress disruption of biological rhythms and the onset of diseases due to insomnia; however, chronic use of these drugs show many side effects due to resistance and dependence [2,3]. Therefore, various studies have been conducted to screen and utilize natural substances that not only alleviate and treat insomnia but also have no side effects. Jujube (*Ziziphus jujube*), Hope (*Humulus lupulus*), Valerian (*Valeriana officinalis* L.), and lotus (*Nelumbo nucifera* Gaertn) extracts are natural substances and are used worldwide owing to their relaxation and sleep-improving effects as well as safety [3,4,5,6].

Lotus is widely used throughout East Asia, and all parts of lotus, including root, seed, seed pod, flower, and leaf, are used for ornamental and food purposes, including tea [7]. It has been reported that gamma-aminobutyric acid (GABA) and alkaloids contained in lotus seeds and leaves exert sedative and sleep effects via GABA_A_ receptor binding. Alkaloids, such as romerine, nuciferine, armepavine, N-nornuciferine, and pronuciferine, contained in lotus leaves have a sedative effect in the central nervous system; indeed, they are used as natural substances for relieving insomnia [8]. Lotus leaves also contain a high content of flavonoids, such as quercetin-3-glucuronide (Q3G), quercetin-3-*O*-glucoside, and kaempferol-3-*O*-glucoside, quercetin, which have various biological activities such as tumor-inhibitory, vasorelaxant, and antioxidant activities [9,10].

Q3G, also known as miquelianin, is a flavonol glucuronide present in wine [11], St John’s wort [12], green beans [13], and lotus [14]. Previous in vitro studies demonstrated that Q3G can reach the central nervous system from the small intestine [15]. The physiological activity of Q3G contained in the herbal plant extract is being actively researched. Q3G significantly reduces β-amyloid peptide generation by primary neuron cultures generated from the Tg2576 Alzheimer’s mouse model [16]. Q3G induces the proliferation and migration of neural stem cells through the Akt/cyclin D1 and brain-derived neurotrophic factor signaling pathway [17]. In addition, Q3G was found to have sedative, anxiety-relieving, and anticonvulsant effects by binding to GABA receptors [18]. However, the effect of lotus leaves containing Q3G on the sleep–wake cycle and sleep quality remains obscure. Therefore, in this study, the effect of lotus leaf extract containing Q3G on the sleep cycle was investigated along with the neural mechanisms associated with sleep-improving activity of Q3G.

## 2. Results

### 2.1. Extraction Yield and Concentrations of Carbohydrates, Amino Acids, Polyphenols, Flavonoids, and Q3G in the Ethanolic Lotus Leaf Extract

The concentrations of carbohydrates, amino acids, flavonoids, and polyphenols in the ethanolic lotus leaf extract are shown in Table S1. Analysis of the ethanolic lotus leaf extract showed that the extraction yield was 8.95 g/100 g of raw lotus leaf, and remaining 90% were summed to non-soluble compound into 70% ethanol solvent. The highest concentration was that of polyphenols (285.48 μg/mg), also the concentrations of total carbohydrates, amino acids, and flavonoids were 156.31 μg/mg, 46.55 μg/mg, and 33.13 μg/mg, respectively. The concentration of polyphenols in the ethanol extract was approximately over 30.0%, which was significantly higher than that of carbohydrates, proteins, and flavonoids (Table S1). 

### 2.2. Effects of the Ethanol Extract of Lotus Leaf on Sleep Latency and Duration

**Figure 1:**
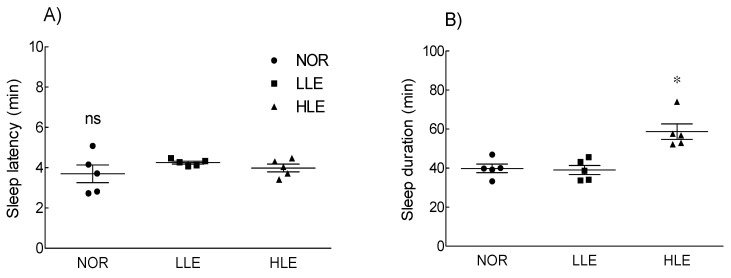
Effects of the ethanol extracts of lotus leaf on (**A**) sleep latency and (**B**) sleep duration in mice (*n* = 5/group) intraperitoneally injected with a hypnotic dose of pentobarbital (42 mg/kg). NOR: normal group; LLE: low dose of ethanol-extracted lotus sample-treated group (150 mg/kg); HLE: high dose of ethanol-extracted lotus sample-treated group (300 mg/kg); ns, not significant. Values are means ± standard error of the mean for each group. * *p* < 0.05 vs. NOR.

The effect of the ethanolic lotus leaf extract on sleep latency and duration is shown in Figure 1. Sleep latency was not significantly different between the treatment groups (low dose of lotus extract [LLE]: 150 mg/kg; high dose of lotus extract [HLE]: 300 mg/kg) and the NOR group. However, the HLE group (53.52 ± 2.52 min) had a significantly longer sleep duration than that of the NOR group (40.0 ± 3.85 min).

### 2.3. Effects of the Ethanolic Lotus Leaf Extract on Sleep Architecture

**Figure 2:**
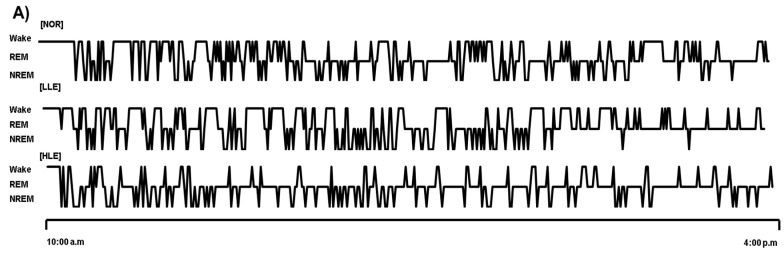

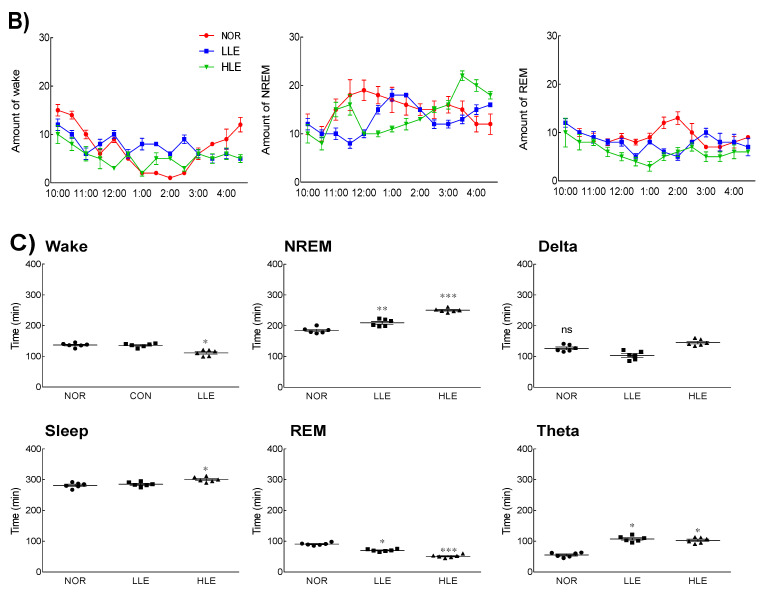
Effects of the ethanol extracts of lotus leaf on sleep architecture in rat (*n* = 6/group). (**A**) Hypnograms that represent concatenated 1 min epochs of EEG activity, scored as wake, rapid eye movement sleep (REM), non- rapid eye movement sleep (NREM) sleep on day 9th. (**B**) Changes on wake, REM, NREM after oral injection for 7 h (**C**) Average time of awakening, sleep, non-REM, REM, delta waves, and theta waves in SD rats. NOR: normal group; LLE: low dose of ethanol-extracted lotus sample-treated group (150 mg/kg); HLE: high dose of ethanol-extracted lotus sample-treated group (300 mg/kg); REM: rapid eye movement sleep; NREM: non-REM sleep; ns, not significant. Values are means ± standard error of the mean for each group. * *p* < 0.05, ** *p* < 0.01, and *** *p* < 0.001 vs. NOR.

Figure 2 shows the effect of the ethanolic lotus leaf extract (150 and 300 mg/kg) on sleep architecture. To evaluate its sleep quantity- and quality-improving effect, rats were administered with the lotus leaf extract prior to commencing the pentobarbital-induced sleep test. Administration of the high dose of the ethanolic lotus leaf extract significantly elevated wake and sleep times (305 ± 10.58 min) compared to the NOR group (284 ± 8.85 min) (Figure 2, *p* < 0.05). Moreover, although ethanol extract administration increased sleep time, both LLE and HLE groups had a significantly lower rapid eye movement (REM) sleep time (LLE: *p* < 0.05, HLE: *p* < 0.001) and significantly higher non-rapid eye movement (NREM) sleep time in a dose-dependent manner (LLE: *p* < 0.01; HLE: *p* < 0.001) than those of the NOR group. Furthermore, among the electroencephalogram (EEG) theta and delta waves, which correspond to NREM sleep, the ethanolic lotus leaf extract significantly influenced the theta waves (*p* < 0.05), but not the delta waves compared to the NOR group (Figure 2).

### 2.4. Effects of the Ethanolic Lotus Leaf Extract on Sleep Architecture in the Caffeine-Induced Insomnia Model

**Figure 3:**
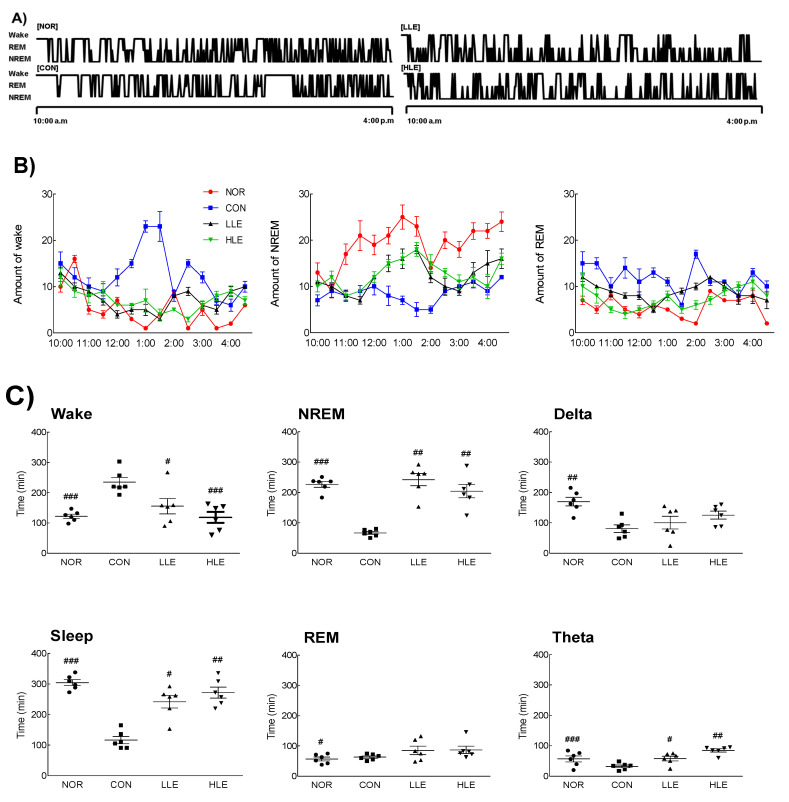
Effects of the ethanol extracts of lotus leaf on electroencephalography of caffeine-induced insomnia model (*n* = 6/group). (**A**) Hypnograms that represent concatenated 1 min epochs of EEG activity, scored as wake, rapid eye movement sleep (REM), non-rapid eye movement sleep (NREM) sleep on day 9. (**B**) Changes on wake, REM, NREM after oral injection for 7 h (**C**) Average time of awakening, sleep, non-REM, REM, delta waves, and theta waves for 9 days. NOR: normal group; CON: caffeine-treated group (80 mg/kg); LLE: low dose of ethanol-extracted lotus leaf-treated group (150 mg/kg) with caffeine; HLE: high dose of ethanol-extracted lotus leaf-treated group (300 mg/kg) with caffeine; REM: rapid eye movement sleep; NREM: non-REM. Values are means ± standard error of the mean for each group. ^#^ *p* < 0.05, ^##^ *p* < 0.01, and ^###^ *p* < 0.001 vs. CON.

Next, we assessed the effect of the lotus leaf extract on the quality and quantity of sleep, particularly the sleep structure, in the caffeine-induced insomnia model (Figure 3). An insomnia model was successfully established, as indicated by a significant difference in wake and sleep times between the caffeine-administered (CON) and NOR groups (*p* < 0.001). In the caffeine-induced insomnia model, the LLE and HLE groups had significantly lower wake times (LLE: *p* < 0.05; HLE: *p* < 0.001) and significantly higher sleep times compared to those of the CON group (LLE: *p* < 0.05; HLE: *p* < 0.01). Contrarily, there were no significant differences in wake and sleep times between the treatment groups and the NOR group. Furthermore, NREM sleep was significantly lower in the CON group than in the NOR group (*p* < 0.001), whereas there was no difference in REM sleep. Similarly, there was no significant difference in NREM sleep between the treatment groups and the NOR group (*p* < 0.01). Moreover, the CON group showed a significant decrease in theta and delta waves, which is related to sleep quality, compared to those in the NOR group (*p* < 0.01, *p* < 0.001, respectively). The LLE and HLE groups had results similar to those of the NOR group.

### 2.5. Effect of Q3G on Sleep Enhancement and Binding Activity to the GABA_A_ Receptor

**Figure 4:**
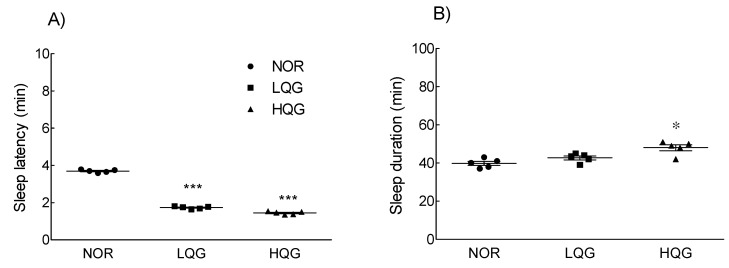
Effects of quercetin-3-*O*-glucuronide on (**A**) sleep latency and (**B**) sleep duration in mice (*n* = 5/group) intraperitoneally injected with a hypnotic dose of pentobarbital (42 mg/kg). NOR: normal group; LQE: low dose of Q3G-treated group (10 mg/kg); HQE: high dose of Q3G-treated group (20 mg/kg). Values are means ± standard error of the mean for each group. * *p* < 0.05 and *** *p* < 0.001 vs. NOR.

**Figure 5:**
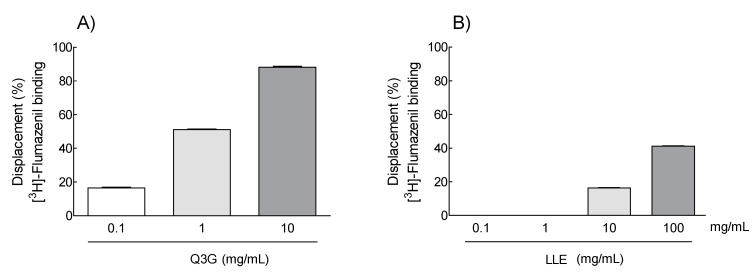
Dose–response displacement (%) of [^3^H]-flumazenil binding by (**A**) quercetin-3-*O*-glucuronide (Q3G: 0.1, 1, and 10 mg/mL) and (**B**) the lotus leaf extract (LLE: 0.1, 1, 10, and 100 mg/mL) was analyzed by GABA_A_-BDZ receptor binding assay. Values are means ± standard deviation for each group.

The concentration of the antioxidant compounds polyphenols and flavonoids in the ethanolic lotus leaf extract was high; therefore, the concentration of Q3G, a type of phenolic compound, was analyzed by HPLC (Figure S1). Q3G concentration in the ethanolic lotus extract was 77.27 μg/mg, which is approximately 8% of the lotus leaf extract (Figure S1). When the dose of the ethanolic lotus leaf extract was 300 mg/kg (Figure 2), which showed a significant difference compared to NOR group, the estimated dose of Q3G was 20 mg/kg, which is approximately 8%, in the pentobarbital-induced sleep test. Furthermore, to evaluate whether Q3G contributes to sleep enhancement, sleep latency and duration were measured in the pentobarbital-induced sleep model (Figure 4). Administration of 10 and 20 mg/kg Q3G showed a significant decrease in sleep latency (*p* < 0.001), while 20 mg/kg Q3G administration significantly increased sleep duration (*p* < 0.05) compared to the NOR group. To evaluate binding activity of Q3G to the GABA_A_ receptor, 0.1, 1, and 10 mg/mL of Q3G solution was applied on purified GABA receptors from homogenized Sprague Dawley (SD) rat brain tissues. Based on the calculation, 20 g of mice (oral dosage 150 and 300 mg/kg) take 3 mg and 6 mg of the extract in a day, and it also means 0.21 mg and 0.42 mg of Q3G. Therefore, from 0.1 mg to 10 mg of Q3G was tested for binding activity, and 0.1 mg to 100 mg of the lotus leaf extract (LLE) was screened in wider concentration. Figure 5 illustrates the binding activity of Q3G to the GABA_A_-BDZ receptor against [^3^H]-Flumazenil binding at the BDZ binding site. The binding activity of Q3G at 10 mg/mL was approximately 92.4% and that at 1 mg/mL was 51%.

## 3. Discussion

Sleep disturbance is commonly treated with drugs such as benzodiazepines and non-benzodiazepines. However, they may cause drug dependence and have long-term side effects such as headache, nightmares, daytime fatigue, nausea, dizziness, and falling. Therefore, development of natural product-derived therapeutics effective against insomnia and with no side effects is crucial. *N. nucifera*, including its leaves, seeds, roots, and flowers, has been used as a herbal remedy to treat and prevent sleep disturbances. However, there are limited studies on the active compounds of lotus or mechanisms involved in improving sleep.

In this study, oral administration of the ethanolic lotus leaf extract was shown to increase sleep time in the pentobarbital-induced sleep model (Figure 1). In addition, as the dose of the ethanolic lotus leaf extract increased, duration of REM sleep decreased and that of NREM sleep increased. The mouse dosage, 150 mg/kg and 300 mg/kg are estimated the human dosage as 12 mg/kg and 24 mg/kg based on human equivalent dose calculation chart [19]. The increase in NREM sleep was attributed to theta waves, which may be due to high concentration of Q3G contained in the lotus leaf extract. Q3G is a compound of flavonol, a glycoside derived from quercetin that exhibits strong antioxidant, anti-inflammatory, inhibition of lipid peroxidation, and anti-obesity effect like quercetin [19]. Plants such as *Polygonum perfoliatum* [20], *N. nucifera* [21], and green beans [13] contain Q3G, which reportedly is the main active ingredient of the water extract of the lotus leaf [22]. Consistently, our results confirmed that Q3G in the ethanolic lotus leaf extract enhanced sleep quantity and quality (Figure 1).

Lotus contains numerous parts: lotus flower, seed pod, seed, leaf, and root [21], and has various physiological activities. The most studied active compounds in lotus leaves are alkaloids such as neferine and nuciferine [23]. Lotus leaf alkaloids are a major component and have shown to promote proliferation of human osteosarcoma cells [24] and exert a sedative effect via the GABA_A_ receptor [8]. Additionally, according to phenolic acids and flavonoids HPLC analysis in lotus leaf extract, the low concentration of catechin (3.69 ± 0.12 μg/mg), caffeic acid (0.02 ± 0.00 μg/mg), and quercetin (0.94 ± 0.05 μg/mg) was detected (Figure S2). However, our study found Q3G as a major component in the ethanolic lotus leaf extract as opposed to alkaloids. Lotus leaf extract containing Q3G not only increased sleep duration in the pentobarbital-induced sleep model, but also influenced sleep quality, as evidenced by EEG test that analyzes specific brain waves in REM and NREM sleep.

The ratio of REM/NREM, including delta and theta waves, is a key factor in determining the quality of sleep [25]. Sleep quality is critically associated with emotional states such as satisfaction, tension, depression, anger, mental fatigue, and confusion. Although REM sleep is important for learning and long-term memory [26], excessive REM sleep causes sleep disturbance, a misalignment of the circadian clock, and is a risk factor for serious diseases such as cardiovascular diseases, diabetes, cancer, and psychiatric disorders [27]. In NREM sleep, theta waves function as a switch for falling asleep and awakening, while delta waves decrease stress and regenerate brain tissue [28]. Therefore, ideally, natural extracts should decrease REM sleep duration and increase NREM sleep time, especially the delta waves. Caffeine acts as an antagonist of adenosine receptors and it commonly results in acute and chronic insomnia, depression, or memory distribution [29]. As caffeine administration decreases delta waves, a caffeine-induced insomnia model can be used to examine sleep regulation.

The possible mechanism of the lotus leaf extract on sleep latency is via GABA receptors on brain tissues. The GABA receptor is the main receptor of sleep function, and scientific evidences using various models have indicated that it is directly related to sleep [30]. Here, we observed a direct binding ability of Q3G to the GABA_A_ receptor by the ligand binding assay, and the lotus leaf extract containing a large amount of Q3G also showed the binding ability to the GABA_A_ receptor. Based on these results, the sleep-enhancing activity of the lotus leaf extract is due to Q3G and its potential interaction with the GABA_A_ receptor. By binding to the GABA_A_ receptor, the permeability of chloride ions though the receptor increases in postsynaptic cells, leading to hyperpolarization and suppression of synaptic transmission. Q3G may indirectly promote GABA neurotransmission by enhancing the permeability of chloride ions, as it binds to the receptor in all parts of the central nervous system. Lotus leaves are rich in alkaloids, and the major compounds are nuciferine, neferine, and pronuciferine [21]. Yan et al. [7] reported that alkaloid extract from lotus leaf exerts its hypnotic and anxiolytic effects through the GABA_A_ receptor [8]. Additionally, neferine contained in lotus seeds presents an anti-depressant activity through hydroxytryptamine (5-HT) metabolism by directly binding to the 5-HT1A receptor as an agonist [31].

Further investigation of the ethanolic extract of lotus leaf to discover other compounds revealed the presence of flavonoids, such as leucoanthocyanidin, quercetin, catechin, and astragalin. Quercetin is a polyphenolic flavonoid and one of major bioflavonoid in the human diet. Quercetin is present in food in glycoside forms, such as quercetin glucoside, galactoside, and arabinoside. The glycoside forms are only partially absorbed in the small intestine and limited quercetin aglycones are detected after consuming quercetin orally. In fact, studies have demonstrated that the levels of quercetin in the lung, kidney, colon, liver, and brain of rats and pigs are low [32]. Moreover, quercetin has a toxicity profile in animal and human studies, which is related to specific health conditions such as cardiovascular diseases, cancer, infections, anti-inflammatory processes, gastrointestinal tract dysfunction, and diabetes [33]. The half-life of quercetin is between 11 and 28 h, suggesting a potential for a significant increase in plasma concentrations upon supplementation. Furthermore, quercetin and its metabolites can pass the blood–brain barrier [34]. Quercetin aglycone undergoes an important and extensive biotransformation reaction by synthesis with uridine diphosphate glucuronic acid to produce Q3G [35].

This study analyzed the changes in sleep quantity and quality by examining sleep latency and sleep time, and Q3G may be a potential major compound in the ethanolic lotus leaf extract (Figure 4). In addition, it was verified by ligand binding analysis that Q3G is involved in GABAergic signaling (Figure 5). Moon et al. [36] reported that Q3G stays in human and rat plasma for a longer time compared to other flavanols because of the catechol structure which makes it stable even after conjugation [37]. Additionally, Ye et al. demonstrated appearance of Q3G after oral administration of 45 mg/kg of lotus leaf extract in both urine and plasma of rat [38]. Q3G transmittance through the blood–cerebrospinal fluid barrier, the glycoside form of Q3G is, therefore, thought to be able to dissolve into the blood and pass directly through the BBB to reach the brain receptor. Additionally, in vitro studies indicated that Q3G reaches the central nervous system from the small intestine [15] and it significantly suppresses reactive oxygen species formation in differentiated neuron cells such as PC-12 cells [39]. However, there are no studies that reveal specific mechanisms related to sleep and Q3G.

Collectively, as a result of investigating the sleep-inducing effect of the ethanolic lotus leaf extract containing Q3G, we confirmed that an increase in sleep time and NREM sleep are mediated by GABA_A_-benzodiazepine receptor binding. To further assess the effect of Q3G isolated from the lotus leaf extract on NREM/REM sleep and specific biomolecules, we aim to perform mechanism studies in the future.

## 4. Materials and Methods

### 4.1. Sample Preparation

Dried Korean lotus leaves were purchased from the local market (Kyungdong Market, Seoul, Korea) and stored at room temperature without light until extraction. Then, 100 g of dried lotus leaves were extracted with reflux using 1000 mL of 70% ethanol at 80–90 °C for 6 h to maximize antioxidant content in the extract [40]. After the extract was centrifuged at 3000× *g* for 20 min, the supernatant was separated using a Whatman NO. 1 filter paper and a vacuum pump. The filtered extract was evaporated with ethanol using a rotary evaporator at 50 °C and stored at −80 °C until use.

### 4.2. Animal Conditions

Male ICR mice and SD rats were subjected to the pentobarbital test and EEG analysis, respectively, for the evaluation of sleep activity. The experiments were approved by the Korean University Institutional Animal Care and Use Committee (KUIACUC-2019-20). Specific-pathogen-free mice (5 weeks old, 18–20 g, *n* = 7/group) and rats (8 weeks old, 160–180 g, *n* = 8/group) were obtained from Oriental Bio Inc. (Seongnam, Korea) and maintained in rooms where light (12 h light/dark cycle), humidity (55 ± 5%), and temperature (22 ± 2 °C) were automatically controlled. Animals had ad libitum access to normal AIN-76A pellet feed (Central Lab. Animal Inc., Seoul, Korea) and water. Three mice and two mice were bred per cage to minimize unexpected aggressive behavior that could occur between individuals. All animals were used for experiments after a one-week adaptation period and were grouped randomly based on average of body weight for each group.

### 4.3. Pentobarbital-Induced Sleep Test

Sleep induction experiment was performed using pentobarbital. To evaluate the intrinsic activity of the extracts, all mice were starved for 24 h. Additionally, mice administered intraperitoneally with pentobarbital were separated and observed in individual cages to minimize unnecessary factors that interfere with sleep activities, such as movement and sound. All extracts were dissolved in 0.9% physiological saline, and ethanol extracts (150 and 300 mg/kg) were orally administrated (*n* = 7/group). After 40 min, hypnotic dose of pentobarbital (42 mg/kg) was intraperitoneally injected to induce sleep, and sleep latency (time to fall sleep) and sleep duration (hours slept) were recorded [41].

### 4.4. EEG Analysis

In order to check the effect of the extract on the frequency of brain waves during sleep, an EEG analysis was performed on SD rats. During surgery, rats were anesthetized with isoflurane (Troika Pharmaceuticals Ltd., Gujarat, India) in a mixture of oxygen and nitrous oxide (7:3 ratio) and then placed in a stereotaxic instrument (Stoelting Inc., Wood Dale, IL, USA). The EEG electrodes (emka Technologies, Paris, France) were fixed to the surface of the skull with prepared dental cement (AgnThós AB, Lidingö, Sweden). After surgery, rats were treated with antibiotics to prevent infection at the surgical site and individually placed in a clean cage to allow them to recover for seven days under the supervision of a veterinarian. The rats were monitored during the recovery period and divided into NOR and treatment groups (*n* = 8/group). In the treatment groups, nocturnal rats were orally administered with the ethanolic lotus leaf extract (150 [LLE] and 300 [HLE] mg/kg) 1 h before conducting the analysis, and the effect of the average sleeping time (11:00 to 18:00) on EEG changes was measured on day 9.

Additionally, to investigate the effect of the ethanolic lotus leaf extract on the caffeine-induced insomnia model, the reduction of caffeine-induced arousal effect was analyzed by oral administration of the extract and 40 mg/kg of caffeine. Cortical EEG signals were acquired using Iox2 (version 2.8.0.13, emka Technologies, Paris, France). The brain wave patterns, wake time, and sleep time were analyzed using EEG spectra and fast Fourier transform of the ecgAUTO3 program (version. 3.3.0.20, emka Technologies, Paris, France) [42]. The different frequencies used for EEG analysis were as follows: delta (δ) wave, 0.5–3 Hz; theta (θ) wave, 3–6 Hz; alpha (α) wave, 6–15 Hz; beta (β) wave, 15–30 Hz; gamma (γ) wave, 30-50 Hz and corresponded to REM (alpha + beta), AW (awake, beta + gamma), SW (slow wave, delta), and QW (quick wave, theta). The pattern of wake, REM, and NREM sleep was visualized as hypnograms that represent concatenated 1 min epochs of EEG activity on day 9th.

### 4.5. Phytochemical Component Analysis of the Ethanol Extract of Leaf

The concentrations of carbohydrates, polyphenols, flavonoids, and Q3G in lotus leaves were determined. Carbohydrate concentration was determined by the sulfuric acid method with some modifications [43], and D-glucose was used as the standard. The total polyphenol concentration was measured by the Folin–Ciocalteu method [44], using gallic acid as the standard, and the total flavonoid content was measured by the *p*-(dimethylamino)cinnamaldehyde method [45], using catechin as the standard.

Q3G analysis was performed using the 1260 series Agilent HPLC system (Agilent Technology, Santa Clara, CA, USA) equipped with the YMC-Trait C18 column (3.9 mm × 250 mm) and a UV detector. The flow rate was 0.3 mL/min and the injection volume was 5 μL. The mobile phase was composed of two solvents: 0.5% formic acid in HPLC grade water (solvent A) and 0.5% formic acid in HPLC grade acetonitrile (solvent B). Gradient elution conditions were as follows: 0−2 min, 93−93% solvent A; 2−24 min, 93−85% A; 24−40 min, 85−70% A; 40−48 min, 70−40% A; 48−50 min, 40–40% A; 50−53 min, 40−10% A; 53−54 min, 10−10% A; 54−55 min, 10−93% A; 55−60 min, 93−97% A. The chromatogram of Q3G was analyzed at 280 nm by a UV detector. The concentration of Q3G standards was calculated using the individual calibration curve.

### 4.6. GABA_A_-BDZ Receptor Binding Assay

The binding assay for the GABA_A_-BDZ receptor was performed according to the method described by Risa et al. [46] with slight modifications. The cerebral cortices extracted from the rats were added to 20 mL of 30 mM Tris-HCl buffer (pH 7.4, kept at 4 °C), homogenized, and then centrifuged at 27,000× *g* for 15 min at 4 °C to recover the pellet. The recovered pellets were stored at −70 °C until use. The frozen membrane was thawed and suspended in a binding buffer (500 mL buffer/original tissue [g]) to prepare a tissue solution for the binding assay. A 96-well plate was added with 180 μL of membrane suspension, 10 μL of extract, and 10 μL of [^3^H] flumazenil (1 nM, final concentration) (Ro 15-1788; Perkin Elmer, Ontario, CA, USA) and incubated in an ice bath for 40 min. Then, it was harvested using a glass fiber filter (GF/C, Whatman). The radioactivity of the sample was measured using a Hidex 300SL counter (Hidex, Turku, Finland). Nonspecific binding (NSB) was determined using BDZ (1 μM). Binding displacement was calculated using the following equation:

Binding displacement (%) = [1 − (DPM_Sample_ − DPM_NSB_)/(DPM_TB_ − DPM_NSB_)] × 100

DPM, disintegrations per minute; TB, total binding; NSB, nonspecific binding.

### 4.7. Statistical Analysis

All data were expressed as mean ± standard error of mean, and differences between each experimental group were evaluated by SPSS version 12.0 (SPSS Inc., Chicago, IL, USA). Differences were compared using Dunnett test, and values of * *p* < 0.05, ** *p* < 0.01, *** *p* < 0.001 vs. NOR group, and ^#^ *p* < 0.05, ^##^ *p* < 0.01, ^###^ *p* < 0.001 vs. CON group were considered statistically significant.

## Data Availability

The data that support the findings of this study are available from the corresponding author upon reasonable request.

## Supplementary Materials

The following are available online, Figure S1: Chromatogram of quercetin-3-*O*-glucuronide (Q3G, R.T. 38.9 min) in the ethanolic lotus leaf extract at 10 ppb concentration; Figure S2: Chromatogram of catechin (3.69 ± 0.12 μg/mg of extract, R.T. 13.6 min), caffeic acid (0.02 ± 0.00 μg/mg of extract R.T. 16.2 min), quercetin (0.94 ± 0.05 μg/mg of extract R.T. 24.2 min) in the ethanol lotus leaf extract at 5 ppb concentration; Table S1: Yield and chemical components in the *Nelumbo nucifera* ethanol extract.

## Sample Availability

Samples of ethanolic lotus leaf extract is available from the first author, Singeun Kim.
