# Supplementary material for: Quercetin-3-O-glucuronide in the Ethanol Extract of Lotus Leaf (Nelumbo nucifera) Enhances Sleep Quantity and Quality in a Rodent Model via a GABAergic Mechanism
Source: Molecules. 2021 May 19;26(10):3023. doi: 10.3390/molecules26103023 (PMC8159104; doi:10.3390/molecules26103023)
Supplement: Supplementary file 1 [file molecules-26-03023-s001.zip › molecules-1207967-supplementary.pdf]

Supplementary material

Supplementary tables

Table S1. Yield and chemical components in the *Nelumbo nucifera* ethanol extract

Parts	Yield	Carbohydrates	Amino acids	Flavonoids	Polyphenols
	(g/100g of raw lotus leaf)	(mg/g of lotus extract)			
Leaf	8.95 ± 0.01	156.31 ± 3.44	46.55 ± 0.31	33.13 ± 2.42	285.48 ± 4.93

Supplementary figures

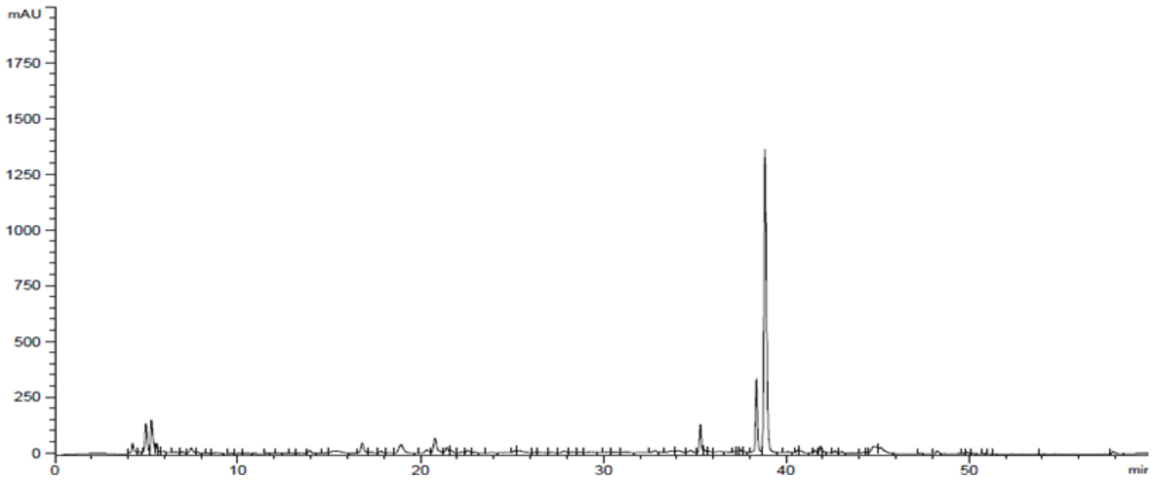

Figure S1. Chromatogram of quercetin-3-O-glucuronide (Q3G, R.T. 38.9 min) in the ethanol lotus leaf extract at 10 ppb concentration

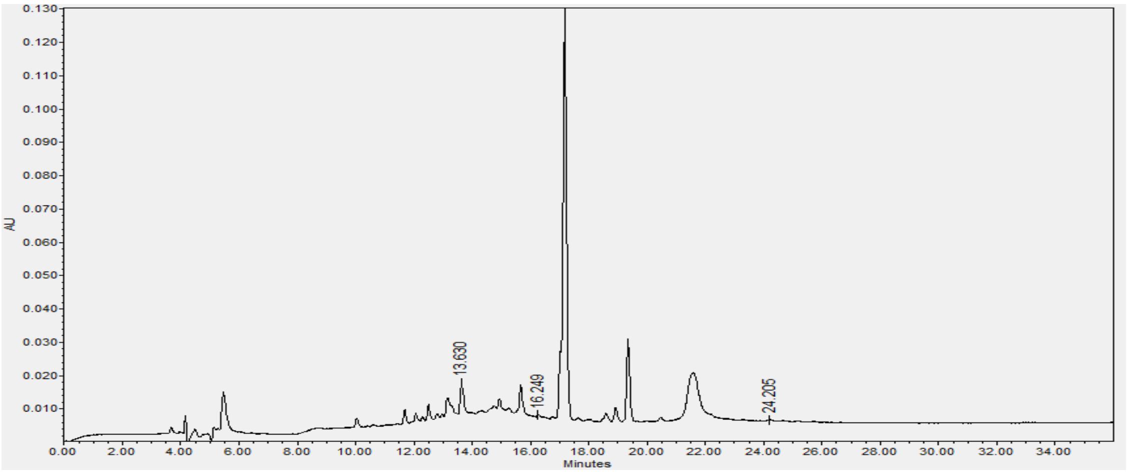

Figure S2. Chromatogram of catechin (3.69 ± 0.12 μg/mg of extract, R.T. 13.6 min), caffeic acid (0.02 ± 0.00 μg/mg of extract R.T. 16.2 min), quercetin (0.94 ± 0.05 μg/mg of extract R.T. 24.2 min) in the ethanol lotus leaf extract at 5 ppb concentration
